# Application of Crude Pomace Powder of Chokeberry, Bilberry, and Elderberry as a Coloring Foodstuff

**DOI:** 10.3390/molecules26092689

**Published:** 2021-05-04

**Authors:** Nicole Jasmin Nemetz, Andreas Schieber, Fabian Weber

**Affiliations:** Institute of Nutritional and Food Sciences, Molecular Food Technology, University of Bonn, Friedrich-Hirzebruch-Allee 7, D-53115 Bonn, Germany; nnemetz@uni-bonn.de (N.J.N.); schieber@uni-bonn.de (A.S.)

**Keywords:** berry pomace, powder, anthocyanins, recovery, sustainability, coloring foodstuff, yogurt

## Abstract

Berry pomace, rich in polyphenols, especially anthocyanins, accumulates during the production of red juices. Pomace from chokeberry (*Aronia melanocarpa* Michx.), bilberry (*Vaccinium myrtillus* L.), and elderberry (*Sambucus nigra* L.) represent good sources of coloring foodstuffs. Pomace powders (PP) were prepared by milling the seedless fractions of the three dried berry pomaces (50 °C, 8 h). Techno-functional properties of the powders such as particle size distribution, bulk density, sedimentation velocity, and swelling capacity were determined to evaluate the powders for possible food applications. Total anthocyanin content was quantified by UHPLC-DAD before and during a storage experiment to monitor the degradation of anthocyanins in the PP and in a yogurt model application. The high content of phenolic compounds and the still intact cell structure ensured high stability of anthocyanins over 28 days of storage. In the model application, color saturation was stable over the whole storage time of 14 days. Regarding the techno-functional properties, only a few differences between the three PP were observed. The particle size of elderberry PP was larger, resulting in lowest bulk density (0.45 g/mL), high cold-water solubility (16.42%), and a swelling capacity of 10.16 mL/g dw. Sedimentation velocity of the three PP was fast (0.02 mL/min) due to cluster formation of the particles caused by electrostatic and hydrophobic properties. Compared to other high-intensity coloring foodstuffs, the use of PP, showing acceptable color stability with potential health-promoting effects, represents a wide applicability in different food applications and especially in products with a longer shelf-life.

## 1. Introduction

In order to preserve the natural color of food, application of artificial dyes has long been common practice. In recent years, the use of these dyes has faced growing concerns due to several adverse effects on human health [1,2,3,4]. Therefore, application of natural food colorants like anthocyanins or carotenoids has been favored. These natural food dyes are obtained from sources like fruits and vegetables by physical processing, generally including separation, pasteurization, and concentration [2,4,5,6]. The intention of adding colorants to food is either to enhance and/or restore natural color, standardize differing batches, or color originally non-colored food [7]. As a consequence of the increasing consumer awareness regarding healthier and more natural food and lifestyle, the food industry seeks to avoid application of food additives mandating the use of E-numbers [4,5,6,8,9]. The labeling of coloring foodstuffs as additives is not compulsory if no selective enrichment of the pigment had taken place [5], rendering coloring foodstuffs very attractive ingredients.

Concentrates from black carrot [10] and purple sweet potato [11] are some of the most abundant coloring foodstuffs. The applicability of anthocyanins, not only in isolated form but also in such concentrates, is limited due to their low stability toward light, temperature, oxygen, pH changes, heat, and other storage conditions [5,8,12,13].

Because of the high instability, their application as microencapsulated powders has become a possible way to improve color properties and also to maintain the potential health benefits of anthocyanins [6,8,13,14].

To fulfill consumer demands regarding naturalness and vivid, diverse colors of food, the industry requires a high variety of coloring foodstuffs from many different sources. One of the upcoming trends is the use of berry pomaces because they are rich in anthocyanins and further bioactive compounds showing potential health-promoting effects [15,16,17,18].

Red berry fruits represent a good source for obtaining anthocyanin-rich spray-dried powders. During fruit juice processing, the pomace accumulates as a by-product. It contains high amounts of valuable components [15,16,17,19,20] such as phenolic compounds which are located in the skin and seeds [21,22]. Several berries contain very high amounts of anthocyanins but a great share of these are not extracted during juice production [23]. The remaining pomace consequently can be used as a good source for these pigments [21,24].

The transformation of by-products into valuable products and food ingredients should be conducted rapidly to ensure a safe and stable product [9,18,25]. The first step of the valorization process is drying of the pomace. This is followed by separation into the seeds and a seedless fraction for further processing into valuable seed oils and powders rich in phenolic compounds [18,26]. Subsequent processing steps of the seedless fraction commonly include solvent extraction, resulting in extracts that may be used as bread additives, in beef, or in cereal-based products [16,18,24,25,26,27]. The residual fiber of pomaces is applied in bakery products to reduce the amount of flour and to increase the dietary fiber content [19]. The incorporation of berry pomace, or parts of it, contributes to the enhancement of nutritional and sensory properties of food [9,15,28].

The present study demonstrates the production of pomace powder from chokeberry (*Aronia melanocarpa* Michx.), elderberry (*Sambucus nigra* L.), and bilberry (*Vaccinium myrtillus* L.) for their application as a coloring foodstuff. Anthocyanin degradation and changes in color were determined during storage and in a model application. Moreover, techno-functional properties of the powders were investigated. The determination of these characteristics of pomace powder will facilitate its food application.

## 2. Results and Discussion

### 2.1. Techno-Functional Properties of Pomace Powder (PP)

Drying of the three different berry pomaces at 50 °C (0% rH, 8 h) followed by the milling of the seedless fraction resulted in fine pomace powders (PP) of chokeberry, bilberry, and elderberry. Techno-functional characterization of these PP is presented and discussed in the following.

Dry matter of the berry pomace and the mass balance during the valorization process of the pomace to PP is shown in Table S1 (supplement material).

#### 2.1.1. Moisture Content and Microbiological Status of the Pomace Powders

The residual moisture content of the three PP was less than 5.30% (Table 1). Significant differences were determined between elderberry PP and the other two PP. Such low residual moisture content was not determined for apple, carrot, and beetroot pomace powders dried at 50 °C to 65 °C for 6–7 h [29] or red grape pomace powder dried at 50 °C. Those powders resulted in a residual moisture content between 5 and 10% [30].

Microbiological status was determined by total bacterial count on PC and YGC culture medium. Results showed overall low colony-forming units (<1 × 10^3^ CFU/g) except for elderberry PP. Elderberry PP showed higher colony-forming units for total bacterial count (Table 1) but did not exceed the standard value of 1 × 10^7^ CFU/g considering microbiological safety [31]. The values were even lower compared to results from Reißner et al. [15]; the lowest values were 1.6 × 10^3^ CFU/g for berry pomace powders produced from the whole pomace and dried at 60 °C for 24 h.

#### 2.1.2. Particle Size Distribution and Morphology

**Figure 1:**
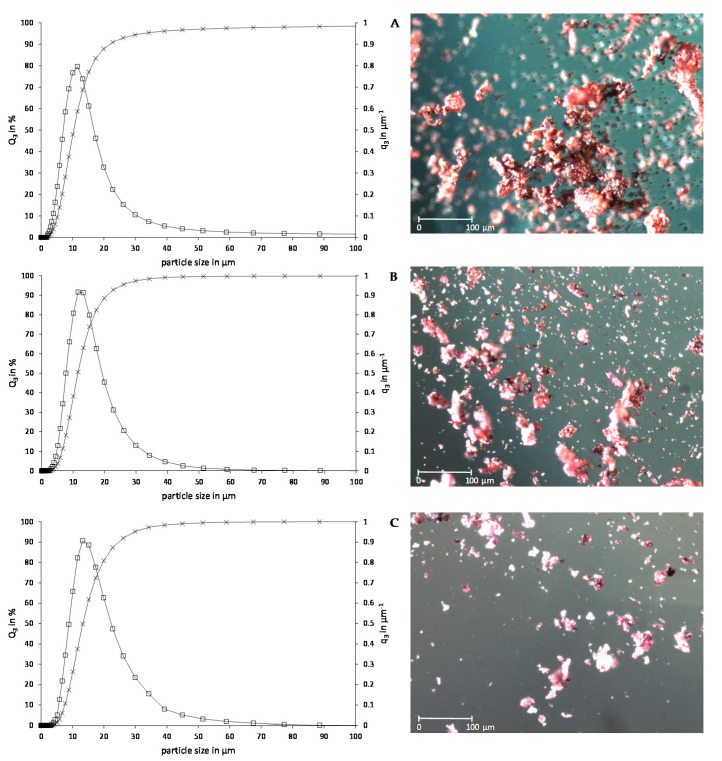
Particle size distribution plotted as the cumulative Q3 in % (×) and density distribution q3 in µm^−^^1^ (□) over particle size in µm and light microscope pictures of chokeberry (**A**), bilberry (**B**), and elderberry (**C**).

Figure 1 shows the particle size distribution and light microscope pictures of PP obtained from drying and milling of chokeberry, bilberry, and elderberry pomace, respectively. Milling of the seedless fraction of dried pomace resulted in fine powders with a mean particle size d_50_ of 10.36 µm (chokeberry), 11.53 µm (bilberry), and 13.28 µm (elderberry) (Table 2). Similar results for d_50_ of 14.55 µm were obtained for red grape pomace powder dried at 50 °C [30]. Larger particles (86–112 µm) were obtained by Reißner et al., who prepared powders by milling the whole pomace after drying at 60 °C for 24 h in an ultra-centrifugal mill [15]. Powders obtained from milling dried apple, carrot, and beetroot pomace in a domestic grinder followed by sieving through a sieve of 250 µm particle size showed particle sizes below 150 µm [29]. The milling conditions have an impact on the size of the particles, resulting in the differences between the compared studies.

Despite similar milling conditions, the d_50_ differed significantly which may be explained by the different fruit structure of the seedless fraction of the dried pomace. The constitution of this fraction of berry pomace caused the heterogeneous particle size distribution of the three PP. With regard to the 90% percentile d_90_, differences between elderberry and the other two were more prominent.

Compared to spray-dried powders, the particle size of all PP is in the lowest quantile of the range of values [32].

Light microscope pictures of the three PP (Figure 1) show spherically shaped particles with a tendency to cohesiveness and the formation of clusters. Smaller particles appear to adhere to the surface of larger ones. Moreover, PP particles showed coarse surfaces of high heterogeneity in shape and size.

The particle morphology and tendency to form clusters affect several technological properties like bulk density, water-binding and oil absorption capacity, cold-water solubility, and sedimentation velocity, which have an impact on the applicability of the PP.

#### 2.1.3. Hygroscopicity

Moisture absorption (%) after 24 h was comparable between the three PP. Although it was lowest in dry matter, chokeberry PP showed the highest moisture absorption after 24 h among all powders (Table 3). Significant differences in the moisture absorption were determined between chokeberry and bilberry. Chokeberry PP tended to form agglomerates resulting in higher moisture absorption. This behavior was already determined for spray-dried powders [32]. Hygroscopicity also depends on the bulk density of the powder. Densely packed powders, like elderberry PP (Section 2.1.4.), enhance the barrier properties toward water and reduce moisture absorption [14]. Differences between the moisture absorption values of elderberry PP and bilberry PP were not significant, but elderberry showed a denser powder.

#### 2.1.4. Bulk Density

All three PP significantly differed in their bulk density, whereby the values of chokeberry PP and bilberry PP only differed by 0.10 g/mL. Elderberry PP resulted in the densest and largest particles with a bulk density of 0.45 g/mL, although the milling process was similar (Table 3). Comparable results for bulk density were determined for goldenberry waste powder (0.63 g/mL) [33] or for apple, carrot, and beetroot pomace powders which had densities of 0.56 g/mL, 0.52 g/mL, and 0.63 g/mL, respectively [29].

Densely packed powders possess several advantages, like the necessity of smaller containers as well as less gas binding due to limited space between particles, which might delay oxidative reactions.

#### 2.1.5. Water-Binding and Oil Absorption Capacity

PP showed little and insignificant differences in water-binding capacities (Table 3). Water-binding and oil absorption capacities of chokeberry PP were comparable to those described by Reißner et al. [15]. Overall, Reißner et al. found higher values for these techno-functional properties of chokeberry, but still in a comparable range. It has to be considered that these authors obtained chokeberry powder from the whole pomace with broader particle size distribution [15].

Water-binding capacity determined for bilberry PP showed the highest value, which is related to the narrow particle size distribution. The presented results are generally lower compared to previous studies on PP of several fruits and vegetables, whereby some differences in the study design have to be considered. The mentioned studies used the whole pomace, or the particle sizes were larger [29,33,34,35]. Particle size affects the hydration properties of the powders which are related to the preservation of the structure in powders showing large particle size, resulting in higher values for water-binding capacity [29,36].

Oil absorption capacity determined using canola and sunflower oil was not significantly different, and significant differences were observed only between elderberry PP and the other two powders. For elderberry PP, an oil absorption capacity of 2.24 and 2.13 g oil/g dw for canola oil and sunflower oil was determined, respectively (Table 3). The oil absorption capacity depends on the porosity of the generated powder which can be deduced from different properties shown for elderberry PP, especially the swelling capacity (see Section 2.1.8). These findings are similar to those reported by Reißner et al. [15] and are comparable to previous studies on goldenberry waste powder, apple pomace powder, and jaboticaba pomace [33,35].

#### 2.1.6. Cold-Water Solubility

PP proved to have only poor cold-water solubility (Table 3). Compared to spray-dried powders which showed high cold-water solubility of over 80% [32], PP shows low applicability in aqueous solutions. This behavior may be explained by the high amount of insoluble cell wall polysaccharides like cellulose and pectin. The powders have a high hydrophobicity and electrostatic property which further lowers their solubility due to the formation of aggregates. Moreover, the porosity of the particles (Figure 1, light microscope pictures) affects the solubility characteristics.

#### 2.1.7. Sedimentation Velocity

The stability of the PP suspensions was determined by the sedimentation velocity (Table 3). Significant differences were observed between elderberry PP and the other two PP. The high values for sedimentation velocity are related to the low cold-water solubility of the PP. Both properties render the application of PP in drinks with low viscosity challenging. Etzbach et al. described similar observations for spray-dried powders with low cold-water solubility and fast emulsion separation [32].

Although PP is a fine powder, cluster formation is a critical issue and causes the low stability in solutions with low viscosity. Cluster formation caused by electrostatic and hydrophobic properties of the powders favors the fast sedimentation. The surface properties of particles have a strong influence on their behavior in solutions. This was determined in light microscopic pictures of the three PP in dry and wet conditions (Figure 1, light microscope pictures).

#### 2.1.8. Swelling Capacity

Swelling capacity of elderberry PP was significantly higher (10.16 mL/g dw) compared to bilberry PP and chokeberry PP (5.16 and 5.08 mL/g dw, respectively) (Table 3). Swelling capacity of the latter two PP were comparable to results obtained for goldenberry and Mexican apple powder, with values of 5.24 mL/g and 3.2 mL/g, respectively [33,34].

The swelling capacity depends on the surface characteristics of particles, showing pronounced porous particles in elderberry PP. These particles showed less electrostatic properties compared to particles from chokeberry PP and bilberry PP, resulting in remarkable swelling capacity between the three powders. Moreover, the densely packed elderberry PP enhanced the barrier properties toward water, leading to a distinct swelling capacity. These results were also observed in a previous study on fruit pomace subjected to bakery products [36].

### 2.2. Anthocyanin and Total Phenolic Contents in Pomace Powder (PP)

The degradation process of total anthocyanins and total phenolics was compared with the stability of reference substances under the same storage conditions. This comparison was performed to evaluate the resistance of individual berry anthocyanins toward degradation compared to anthocyanins in the PP matrix.

The UHPLC-DAD chromatograms of the untreated pomace and the three PP as well as the yogurt applications are shown in Figures S1 and S2, respectively (supplement material).

#### 2.2.1. Storage Stability of Anthocyanins and Phenolic Compounds in PP

**Figure 2:**
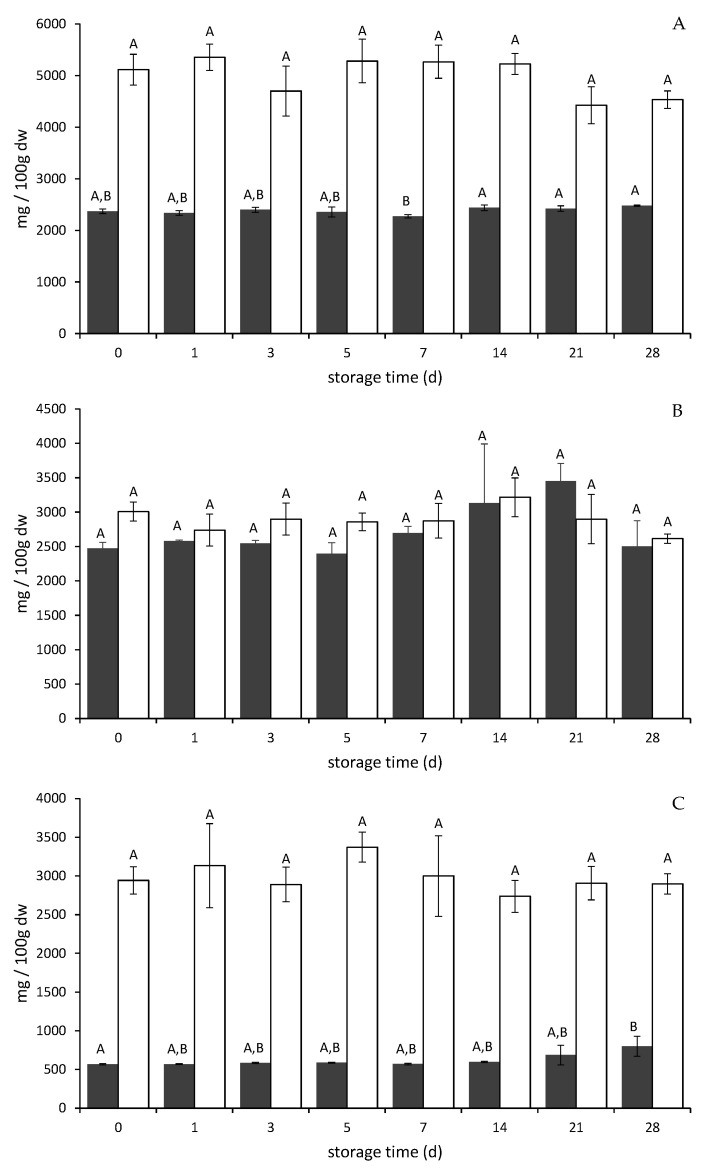
Total anthocyanin content (dark grey) in mg Cya-Glc eq. per 100 g dw of powder and total phenolic content (white) in mg gallic acid eq. per 100 g dw of powder over storage time (in days) of pomace powder of chokeberry (**A**), bilberry (**B**), and elderberry (**C**). Different letters indicate significant differences (*p* ≤ 0.05) within one powder. Values are mean ± standard deviation (*n* = 3).

In Figure 2A–C, the total anthocyanin (dark grey bars) and total phenolic content (white bars) of each PP is shown.

Total phenolic content showed no significant decrease during storage for two PP (Figure 2A,B, white bars) and a constant content for elderberry PP (Figure 2C, white bars).

For chokeberry PP and bilberry PP, the storage experiment revealed a constant total anthocyanin content over 28 days of storage (Figure 2A,B, dark grey bars). The temporal changes during storage were not considerable.

Total anthocyanin content in elderberry PP was constant during the first half of the storage period and increased subsequently with a significant difference compared to the beginning (Figure 2C, dark grey bars). The constant or slightly higher content of total anthocyanins can be related to the strong binding to cell wall components which might attenuate over storage time, rendering anthocyanins more accessible for quantification. It has already been shown that the non-extractable polyphenols, which are associated to cell-wall material, may be released at the end of the storage period [37]. The stability of anthocyanins in the three PP may be explained by the high and constant content of other phenolic compounds and the partly intact cell structures. The gentle drying temperature ensured a PP with high anthocyanin content after processing. Furthermore, elderberries contain anthocyanin diglycosides, which are more resistant towards degradation [38].

Both XAD7 extracts (chokeberry and bilberry) showed comparable results to the PP with constant values of total anthocyanins (supplement material, Table S2). This may be explained by the high amount of other phenolic compounds which act as copigments preventing hydrolysis and oxidation. These protective effects of copigments have already been shown for blackberry XAD7 extract in spray-dried samples [8]. The purified chokeberry and bilberry anthocyanins showed a slight decrease in total anthocyanin content over the storage time, especially in the first half. This may be explained by the removal of stabilizing components during membrane chromatography used for the isolation of anthocyanins.

#### 2.2.2. Color Parameters of the three PP

Color parameters were subjected to considerable changes which are expressed as color difference ∆*E*, hue *h°*, and Chroma C* (Table 4). The highest measured color difference was determined for chokeberry PP with a change of ∆*E* = 3.55 ± 0.03. The three PP differed significantly in their color changes, with the lowest measurable and observed change for bilberry PP (Table 4).

Comparable results were found for the hue angle. The greatest difference was calculated for elderberry PP with a decrease of 2.72 ± 0.19 (Table 4). However, these slight changes in color in storage experiments conducted in the dark were not considerable with respect to the generally intense color of PP. Color changes are not necessarily correlated with the degradation of anthocyanins and vice versa [8], which may be explained by the formation of colored polymers or complexation of several phenolic compounds with other pomace constituents in PP.

A slow but constant release of bound anthocyanins from PP into the surrounding environment can be explained by the techno-functional properties of the three powders and the high hydrophobicity and electrostatic property which hindered hydrolysis and oxidation. This constant release apparently outweighs the degradation of anthocyanins in elderberry PP, resulting in an increase in anthocyanin content at the end of storage.

### 2.3. Anthocyanin and Total Phenolic Content in a Yogurt Model Application over Storage and the Effect on Color Parameters

The degradation process of total anthocyanins and total phenolics was assessed by comparison with the stability of reference substances under the same storage conditions.

Since purple sweet potato anthocyanins and black carrot concentrate were applied in amounts necessary to equate the color saturation of the yogurt with PP, the samples contained only approximately 0.3% of the colorant and coloring foodstuff, respectively.

#### 2.3.1. Storage Stability of Anthocyanins during a Yogurt Model Application

**Figure 3:**
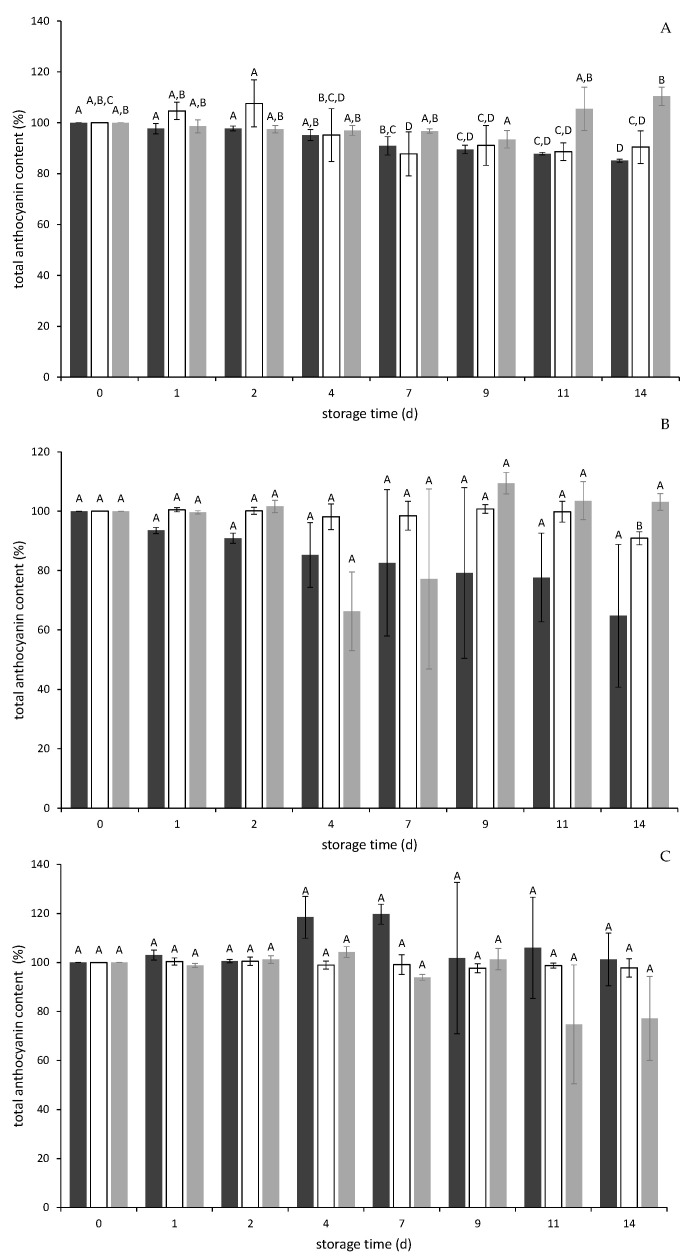
Anthocyanin degradation (%) referred to the initial total anthocyanin content on day 0 in yogurt applications stored for 14 days of pomace powders (dark grey) of chokeberry (**A**), bilberry (**B**), and elderberry (**C**) as well as reference substances, namely purple sweet potato anthocyanins (white) and black carrot concentrate (bright grey). Different letters indicate significant differences (*p* ≤ 0.05) within one yogurt application. Values are mean ± standard deviation (*n* = 3).

For samples containing chokeberry PP and bilberry PP (Figure 3A,B, dark grey bars), total anthocyanins decreased over the storage time with no significant differences between initial total anthocyanin content on day 0 and day 14 (chokeberry: ∆_0–14_total anthocyanin content = −14.89% or 7.07 mg/100g fw yogurt; bilberry: ∆_0–14_total anthocyanin content = −35.21% or 19.98 mg/100g fw yogurt).

In contrast to these changes, the total anthocyanin content of samples containing elderberry PP remained relatively stable with no significant differences over the storage time (∆_0–14_total anthocyanin content = + 1.26% or 0.17 mg/100g fw yogurt) (Figure 3C, dark grey bars). These findings reflected the higher stability of the total anthocyanin content in PP storage experiments (Section 2.2.1). Nevertheless, the high standard deviation on some storage days has to be emphasized. This might be explained by inhomogeneous distribution of the PP and reference substances in the plain yogurt. The homogenization was conducted with gentle agitation to keep the yogurt matrix intact to prevent syneresis.

Compared with the PP yogurt samples, yogurt blended with purple sweet potato anthocyanins showed a constant total anthocyanin content with marginal changes over storage time. Similar results were obtained for black carrot concentrate samples (Figure 3A–C, white and light grey bars). Purple sweet potato and black carrot contain acylated anthocyanins, which have been shown to be more stable than non-acylated derivatives. These acylated anthocyanins are more stable towards temperature, changes in pH, and can be stabilized inter alia by intramolecular copigmentation [10,11].

It should be noted that the absolute anthocyanin content of the reference substances in the yogurt applications was much lower compared to those of PP because a great part of the anthocyanins in PP are bound to the still intact cell structures and are released only slowly during storage. It has to be considered that there is an inhomogeneous dispersion of anthocyanins between the yogurt matrix and the PP particles. Bound anthocyanins might contribute less to the overall color than those that are already released into the yogurt. This might explain the discrepancy between anthocyanin content and color yield.

#### 2.3.2. Storage Stability of Phenolic Compounds during a Yogurt Model Application

The total phenolic content remained constant with no significant differences in bilberry PP and chokeberry PP and increased slightly in elderberry PP samples (Table 5). Comparable results were published for yogurt fortified with freeze-dried grape pomace powder and a storage period of 21 days [39].

Compared with the PP, yogurt applications blended with purple sweet potato anthocyanins and black carrot concentrate showed for two trials a non-significant increase in total phenolic content. The difference in total phenolic content between day 0 and day 14 was more pronounced in the reference substances than in the PP.

The stability of anthocyanins in the three yogurt blends with PP can be explained by the high content of phenolic compounds and the partly intact cell structures. An increase in total phenolic compounds was previously described [40]. The increase can be explained by the limitations of the Folin-Ciocalteu assay used for the analysis as well as by the partial hydrolysis of lactose forming reducing sugars during the fermentation. Additionally, a continuous release of pomace polyphenols over the storage period was shown [40]. These non-extractable polyphenols, which interact with cell wall material, may be gradually released during storage. This may contribute to the increase in total phenolic compounds during the 14 days of storage.

Although the method is generally not well suited for comparing phenolic content of different samples, it can be assumed from Table 5 that the PP-colored yogurts contained considerably higher amounts of phenolic compounds compared to the reference yogurts with purple sweet potato anthocyanins or black carrot concentrate.

#### 2.3.3. Color Parameters of the Yogurt Model Applications

Color parameters determined for each yogurt application resulted in measurable changes over the storage time. Color saturation in yogurt containing PP did not change considerably and decreased only for chokeberry PP and bilberry PP. Samples containing elderberry PP even showed an increase in Chroma (Table 6). Similar changes were observed for the color difference and hue angle. Color stability with slight changes was previously observed for yogurt fortified with freeze-dried grape pomace powder [39].

In contrast, both reference substances showed considerable changes in color parameters compared to the PP. Especially the color of the yogurt colored with purple sweet potato anthocyanins remained less stable and showed a strong decrease in saturation over the storage time (Table 6). This argues for a lower applicability compared to PP due to the strong decrease in color saturation over storage time. Similar results were determined for croissants fortified with 4% elderberry juice. The product provided the same benefits compared to those with the addition of black carrot commercial dye [41].

The powder properties and the surface composition have an influence on the release of anthocyanins. High hydrophobicity and incorporation capability of PP influenced the release of anthocyanins into the yogurt followed by lasting color saturation. Visual appearance was additionally influenced by light scattering effects caused by the particles.

## 3. Materials and Methods

### 3.1. Materials

#### 3.1.1. Chemicals and Standards

Ultrapure water was obtained from a PURELAB flex 2 water purification system (ELGA LabWater, Paris, France). Anthocyanin extraction and analysis were conducted using methanol (HPLC grade) from Fisher Scientific GmbH (Schwerte, Germany), acetic acid glacial (ACS reagent 99.9%), and acetonitrile (LC-MS grade, 99.9%) all from VWR International GmbH (Darmstadt, Germany), formic acid 99.9% (Merck KGaA, Darmstadt, Germany), ethanol 99% (denatured with benzene, Julius Hoesch GmbH, Düren, Germany), and cyanidin-3-*O*-glucoside > 97% (Phytoplan, Heidelberg, Germany). *n*-Hexane (VWR International GmbH, Darmstadt, Germany) and Span^®^ 65 (Merck KGaA, Darmstadt, Germany) were used for particle size distribution analysis. Total phenolic content was determined using Folin-Ciocalteu’s phenol reagent (Merck KGaA, Darmstadt, Germany), sodium carbonate > 99% (Carl Roth, Karlsruhe, Germany), and gallic acid 98% (Alfa Aesar, Kandel, Germany). Sodium chloride ≥ 99.5% (Carl Roth, Karlsruhe, Germany) was used for the determination of the hygroscopicity. Purple sweet potato anthocyanins were obtained from Vitiva d.d. (Markovci, Slovenia), and black carrot concentrate was kindly provided by Döhler GmbH (Darmstadt, Germany). Plain yogurt (3.8% fat), canola, and sunflower oil were purchased from a local supermarket.

Anthocyanin powder reference substances—namely chokeberry and bilberry XAD7 extracts and their further purified anthocyanin extracts—were isolated as described by Larsen et al. [42]. Anthocyanins were extracted at ambient temperature from chokeberry and bilberry juice provided by Haus Rabenhorst O. Lauffs GmbH & Co. KG (Unkel, Germany). The XAD7 extracts were obtained using column chromatography with Amberlite XAD7 HP (Sigma-Aldrich, Munich, Germany). The polyphenols were eluted with ethanol/acetic acid (19:1, *v*/*v*) and subsequently lyophilized. The resulting XAD7 extract was further purified by membrane chromatography with a membrane adsorber Sartobind S IEX 150 mL (Sartorius Stedim Biotech, Göttingen, Germany) obtaining the chokeberry and bilberry anthocyanins.

#### 3.1.2. Berry Pomace as a Raw Material for the Production of Pomace Powder (PP)

The berry pomaces (*Aronia melanocarpa* Michx., chokeberry; *Vaccinium myrtillus* L., bilberry; *Sambucus nigra* L., elderberry) were kindly provided by Haus Rabenhorst O. Lauffs GmbH & Co. KG (Unkel, Germany) and were stored in 5 kg batches at −20 °C until further use. Samples of approximately 100 g were thawed overnight (at 20 °C) in an aluminum bowl covered with a cling film. After thawing, the pomace was dried in a constant climate chamber (KBF P, Fa. BINDER GmbH, Tuttlingen, Germany) for about 8 h (50 °C, 0%rH) under manual stirring every 30 min. After drying to a residual moisture content of approx. 5%, the individual batches of each berry were pooled. The drying procedure was repeated three times resulting in 2.4 kg, 4.4 kg, and 3 kg dried berry pomace from bilberry, elderberry, and chokeberry, respectively. Dried material was subjected twice to a malt grinder (grinder size: 0.635–2.54 mm, Brewferm, Brouwland, Beverlo, Belgium) followed by sieving of the pomace into five fractions for elderberry and chokeberry and six fractions for bilberry. The sieve tower (100% intensity, 10 min, Retsch Technology GmbH, Haan, Germany) was built up with sieves of size (mm) 2.5, 2, 1, 0.5, 0.355, and base for bilberry; sieves of size (mm) 2.5, 2, 1, 0.71, and base for elderberry; and sieves of size (mm) 2, 1, 0.71, 0.5, and base for chokeberry. The seedless fraction was obtained from the base sieve in the case of chokeberry and bilberry and from the 0.71 mm sieve and base sieve for elderberry. Other fractions were the seed fraction on sieves (mm) 0.5 and 0.355 for bilberry, 0.71 and 0.5 for chokeberry, and 1 for elderberry. The agglomerate fraction was located on sieves (mm) 2.5 and 2 for bilberry, 2 and 1 for chokeberry, and 2.5 and 2 for elderberry. The seedless fraction was pulverized in a vibratory disc mill, type RS200 (Retsch Technology GmbH, Haan, Germany) at 14,000 rpm for 40 s (ambient temperature). The resulted powders were stored at −20 °C until further use.

### 3.2. Methods

#### 3.2.1. Storage of Pomace Powder (PP)

To evaluate the stability of the produced PP of chokeberry, bilberry, and elderberry, approximately 10 g of each PP were packed in eight vacuum bags, sealed (vacuum sealer, Vac-Star 2000GSL, Bern, Swiss), and stored at elevated temperature (35 °C, 0% rH, dark) in a constant climate chamber (KBF P, Fa. BINDER GmbH, Tuttlingen, Germany) for a period of 28 days to simulate prolonged storage. Samples were taken after 1, 3, 5, 7, 14, 21, and 28 days. As references during storage of PP, chokeberry and bilberry XAD7 extracts (approximately 1 mg) and the corresponding purified anthocyanin extracts (approximately 0.5 mg) were packed in eight vacuum bags each (see above) and subjected to the same storage conditions. After sampling, PP and reference bags were stored at −80 °C until analysis.

#### 3.2.2. Storage of Colored Yogurt

To evaluate the applicability of the produced PP of chokeberry, bilberry, and elderberry, the three powders (6 g) were mixed manually into approximately 315 g plain yogurt (2% PP, *w/w*). Followed by transferring approximately 35 g yogurt sample into eight 50 mL sealable test tubes, the head space was flushed with N_2_ and sealed with Parafilm^®^ M. Yogurt samples were stored at 4 °C in a constant climate chamber in the dark at 0% rH (KBF P, Fa. BINDER GmbH, Tuttlingen, Germany) for 14 days, with samples taken on days 1, 2, 4, 7, 9, 11, and 14. After sampling, yogurt samples were stored at −80 °C after spectrophotometrical determination of CIE L*a*b* parameters.

As a comparison, yogurt was colored with two reference substances which were the frequently used colorant from purple sweet potato and the coloring foodstuff from black carrot concentrate. The concentration was set to adjust the color saturation (Chroma, C*) similar to each PP (Table 7). The mixing and test tube preparations were conducted according to the yogurt samples blend with PP.

#### 3.2.3. Extraction and Quantification of Anthocyanins in Stored PP and Yogurt

Anthocyanin extraction and quantification from PP and yogurt samples were performed as reported previously by Heffels et al. [43]. Extraction solvent with a ratio of methanol/water/acetic acid (80:15:5, *v/v/v*) was used. Lyophilization prior to extraction was omitted. Anthocyanins were extracted in triplicate by weighing approximately 1.2 g PP or 10 g yogurt samples in test tubes. Powder samples were homogenized with an Ultra-Turrax, and yogurt samples were vortexed. Centrifugation was carried out at 4 °C (11,000× *g*, 10 min). The extraction procedure was repeated twice, first with 20 mL extraction solvent and after transferring the supernatant into a 50 mL volumetric flask, the pallet was extracted again with 10 mL extraction solvent. After the second centrifugation (4 °C (11,000× *g*, 10 min), both supernatants were pooled and made up to the mark with water.

Ultrahigh-performance liquid chromatography diode array detector (UHPLC-DAD) analysis was performed on a Prominence UFLC system (Shimadzu, Kyoto, Japan) equipped with two Nexera X2 LC-30AD high-pressure gradient pumps, a Prominence DGU-20A5R degasser, a Nexera SIL-30AC Prominence autosampler (15 °C, injection volume 5 μL), a CTO-20AC Prominence column oven (40 °C), and a SPDM20A Prominence diode array detector. Data acquisition and processing were performed using LabSolutions software version 5.85 (Shimadzu, Kyoto, Japan). Anthocyanin separation was carried out on an ACQUITY UPLC HSS T3 column (2.1 μm, 150 × 1.8 μm; Waters, Milford, MA, USA) as well as on a Kinetex C-18 column (1.7 μm, 150 × 2.1 mm, Phenomenex, Inc., Aschaffenburg, Germany) equipped with a security guard cartridge of the same material (2.1 × 5 mm, 1.7 μm). The following gradient was used where eluent A was water/formic acid (97:3, *v/v*) and eluent B was acetonitrile/formic acid (97:3, *v/v*) at a flow rate of 0.4 mL·min^−1^/(min/% B): 0/4, 2/4, 7/8, 13/10, 19/17, 23/30, 23.3/100, 25.3/100, 25.8/4 and 0/4, 2/4, 5.5/6, 13/8, 18/9, 23/14, 25/30, 25.3/100, 27.3/100, and 27.8/4 for HSS T3 and Kinetex, respectively. Anthocyanins were detected at 520 nm and quantified as cyanidin-3-*O*-glucoside equivalents (Cya-Glc eq.) by external calibration. Total anthocyanin content was calculated as the sum of individual anthocyanins. Analytes were identified by comparing elution order and UV/Vis spectra with those in previous studies [42,43,44,45].

The HSS T3 column was used for all samples containing anthocyanins from chokeberry, namely all PP of chokeberry, the XAD7 extracts of chokeberry, and the corresponding purified anthocyanin extracts.

All other samples containing anthocyanins from elderberry or bilberry were analyzed on the Kinetex C-18 column.

Purple sweet potato anthocyanin and black carrot concentrate samples were analyzed on both columns to compare the total anthocyanin content with each berry due to the use of two columns. Values measured on HSS T3 were compared with the values for chokeberry samples, and values from Kinetex C-18 were compared with samples from bilberry and elderberry.

#### 3.2.4. Determination of Total Phenolic Content by the Folin-Ciocalteu Assay

For the determination of total phenolic content, sample solutions of PP and the yogurt samples were prepared. Therefore, a 0.1% PP in ethanol/water solution (50/50, *v/v*) was centrifuged and the obtained supernatant was used for further analysis of PP samples. Then, 1 mL of extracts obtained from anthocyanin extraction (Section 3.2.3) of yogurt samples were used for the Folin-Ciocalteu assay. Results were expressed as mg gallic acid equivalents/100 mg dw (mg GAE/100 mg dw) by external calibration.

Total phenolic content in PP and yogurt samples was determined as reported previously [46,47,48] with some modifications. First, 840 µL ultra-pure water and 10 µL sample solution were mixed with 50 µL Folin-Ciocalteu reagent in a semi-micro cuvette (1 cm). After 3 min (ambient temperature), 100 µL saturated sodium carbonate solution was added, mixed, and left for 60 min (ambient temperature). After incubation, the absorbance at 720 nm was measured in triplicate runs using a Genesys 6 spectrophotometer (Thermo Fisher Scientific, Waltham, MA, USA).

#### 3.2.5. Determination of Techno-Functional Properties of PP

The techno-functional properties of the processed PP were determined to evaluate the powders for possible food applications.

##### Particle Size

Particle size distribution of the PP was analyzed using a laser scattering particle size distribution analyzer (Horiba Scientific Partica LA-960, Retsch Technology GmbH, Haan, Germany). Powder was dispersed in 0.1% Span^®^ 65 *n*-hexane solution and subjected to the measuring chamber. Cumulative and density distribution as well as particle parameters such as d_10_, d_50_, and d_90_ were determined sixfold.

Particle morphology (surface, cluster formation, and structure) of the powders were assessed with a light microscope (AXIO Lab.A1, Carl Zeiss AG, Oberkochen, Germany) and top light (KL 750, Schott AG, Mainz, Germany) and observed at 10× magnification (0.25 Ph1, N-Achroplan).

##### Hygroscopicity

The hygroscopicity of the PP was determined in triplicate as described by Silva et al. [14] with modifications. Samples were stored in flat aluminum trays in a desiccator and were weighed after 1, 3, 7, and 24 h. Moisture absorption was calculated according to Etzbach et al. [32].

##### Color Parameters (CIELab Color Metrics)

The determination of color parameters (according to CIELab color metrics) of PP and yogurt samples was conducted using a Chromameter CR-400/410 with illuminant D_65_ (Konica Minolta, Langenhagen, Germany). Next, 5 g PP and 35 g yogurt were subjected to a glass cuvette with a diameter of 60 mm. Color loss during storage was calculated as overall color difference ∆*E* according to the equations mentioned previously [8,14] and the readings obtained for color parameters Chroma C* and hue *h°*.

##### Microbiological Status

Then, 1 g of each PP was suspended in 9 mL physiological saline solution and homogenized in blender bags (400 mL, 190 mm × 300 mm, Corning Life Science B.V., Amsterdam, The Netherlands) for 2 min. The solution was decimally diluted (10^−1^–10^−5^) and pour-plated onto a plate count agar as well as on a yeast plate count agar to determine total bacteria counts after 48 h (30 °C) and 3–5 days (25 °C) incubation, respectively.

##### Water-Binding and Oil Absorption Capacity

Water-binding and oil absorption capacity were determined according to Reißner et al. [15] with modifications. Besides canola oil, sunflower oil was also used to determine the oil absorption capacity.

##### Solubility

Cold-water solubility of the three PP was determined in triplicate according to Etzbach et al. [32] with slight modifications. A 50 mL (2%) powder suspension was vortexed for 3 min in a test tube. After centrifugation (11,000× *g*, 4 °C, 10 min), 25 g supernatant was transferred to an aluminum bowl and dried at 110 °C for 24 h. The cold-water solubility was calculated as described previously [32].

##### Bulk Density

Bulk density was determined in triplicate according to Carneiro et al. [49] with some modifications. A sample of 5 g of PP was subjected to a 50:1 mL graduated cylinder and tapped by hand on the lab bench 50 times from a height of 10 cm.

##### Dry Matter

Dry matter (%dw) was determined thermogravimetrically in triplicate using a Sartorius moisture analyzer (MA100Q000230V1, Göttingen, Germany) by drying about 5 g of PP each.

##### Sedimentation Velocity

Sedimentation velocity was determined in triplicate by modifying two published suspension-stability experiments [32,49]. PP (2%, *w/w*) was dissolved in water, and 25 mL of the solution was transferred to test tubes and stored at ambient temperature for one day. The separation (%) was calculated according to Carneiro et al. [49].

##### Swelling Capacity

Swelling capacity was carried out in triplicate following the experimental setup from Reißner et al. [15] with some modifications. First, 0.2 g PP was mixed with 10 mL water for 30 sec in a test tube and placed in a rack for 18 h (ambient temperature). The volume of the swollen powder was determined according to Reißner et al. [15].

#### 3.2.6. Statistical Analysis

Statistical analysis was conducted using XLSTAT software version 2019 (Addinsoft, Paris, France). An ANOVA with Tukey test was performed to determine significant differences (*p* ≤ 0.05). In case the data did not follow a normal distribution, the Kruskal–Wallis test with Dunn test for multiple comparisons was performed.

## 4. Conclusions

Berry pomace, obtained as a side product of juice processing, represents a better raw material for producing an anthocyanin- and phenolic-rich powder compared to the whole fruit. The gentle drying temperature and short time followed by subsequent milling of the seedless fraction of red berry pomace ensured fine, red-colored powders with considerable amounts of anthocyanins and total phenolic compounds without using any solvent or long processing lines. The chokeberry, bilberry, and elderberry pomace powders (PP) showed promising techno-functional properties with the exception of a rather poor applicability in solutions with low viscosity due to a high sedimentation velocity.

Despite the considerably higher dosages, compared to food colorants and coloring foodstuff that are necessary to obtain comparable colors, berry PP still has a great potential as a coloring foodstuff since it represents an inexpensive side-stream product of the berry juice production. The results obtained in the storage experiments suggest the applicability of the three PP, showing acceptable color stability with additional benefits with regard to phenolic compounds. It can be assumed that PP further increases the dietary fiber content in food applications.

The three PP provided the same coloring effect as the purple sweet potato anthocyanins or the black carrot concentrate but presumably added fiber and antioxidative compounds at the same time, raising the potential of PP to be used at industrial level. Compared to other high intensity coloring foodstuffs, the use of PP shows a wide applicability in dry or liquid, savory or sweet, bakery or dairy food applications where the higher amounts are not an issue. Moreover, the release of anthocyanins in PP into the surrounding environment is slow and thus, PP can be used in a wide range of applications especially in products with a longer shelf-life.

Based on the conducted yogurt model application, it is likely that an increased dosage level of PP could influence the techno-functional and sensory properties. Therefore, a validation of the recipe may be conducted to determine the threshold of acceptability. An enhanced standardization of the particle size, obtained by an optimized milling process, might further amend the techno-functional properties.

It might be concluded that the presented strategy can be applied as a valorization process to obtain berry PP suitable to improve the color and the nutritional value of food.

## Figures and Tables

**Table 1:** Moisture content (%) and microbiological status (PC total bacterial count, YGC yeast, and mold count in colony-forming units (CFU)) of pomace powders. Different letters indicate significant differences (*p* ≤ 0.05) within each row. Values are mean ± standard deviation (*n* = 3).

		Chokeberry	Bilberry	Elderberry
Dry matter content (%)		94.70 ± 0.14 ^B^	94.76 ± 0.15 ^B^	96.23 ± 0.36 ^A^
Microbiological status (CFU/g)	PC	<1 × 10^3^	<1 × 10^3^	4.6 × 10^4^
	YGC	<1 × 10^3^	<1 × 10^3^	<1 × 10^3^

**Table 2:** Particle size distribution of pomace powder with the characteristic particle size d_10_, d_50_, and d_90_. Different letters indicate significant differences (*p* ≤ 0.05) within each column. Values are mean ± standard deviation (*n* = 6).

	d_10_ (µm)	d_50_ (µm)	d_90_ (µm)
Chokeberry	5.21 ± 0.04 ^C^	10.36 ± 0.06 ^C^	21.93 ± 0.74 ^B^
Bilberry	6.50 ± 0.46 ^B^	11.53 ± 0.83 ^B^	20.93 ± 2.67 ^B^
Elderberry	7.57 ± 0.41 ^A^	13.28 ± 0.66 ^A^	24.69 ± 1.54 ^A^

**Table 3:** Techno-functional properties of the pomace powders. Different letters indicate significant differences (*p* ≤ 0.05) within each row. Values are mean ± standard deviation (*n* = 3).

	Chokeberry	Bilberry	Elderberry
Moisture absorption after 24 h (%)	4.09 ± 0.13 ^A^	3.31 ± 0.12 ^B^	3.63 ± 0.29 ^A,B^
Bulk density (g/mL)	0.73 ± 0.03 ^A^	0.63 ± 0.00 ^C^	0.45 ± 0.01 ^B^
Water-binding capacity (g water/g dw)	2.43 ± 0.10 ^A^	3.10 ± 0.07 ^A^	2.38 ± 0.76 ^A^
Oil absorption capacity (g canola oil/g dw)	1.74 ± 0.18 ^B^	1.79 ± 0.09 ^B^	2.24 ± 0.10 ^A^
Oil absorption capacity (g sunflower oil/g dw)	1.76 ± 0.08 ^B^	1.85 ± 0.05 ^B^	2.13 ± 0.03 ^A^
Cold-water solubility (%)	10.98 ± 0.03 ^B^	10.98 ± 0.17 ^B^	16.42 ± 0.26 ^A^
Sedimentation velocity (mL/min)	0.02 ± 0.00 ^A^	0.02 ± 0.00 ^A^	0.02 ± 0.00 ^B^
Swelling capacity (mL/g dw)	5.08 ± 0.10 ^B^	5.16 ± 0.10 ^B^	10.16 ± 0.18 ^A^

**Table 4:** Color difference ∆*E*, hue angle *h°,* and Chroma C* of pomace powder of chokeberry, bilberry, and elderberry over storage time and day 0 and day 28. Values are mean ± standard deviation (*n* = 3).

		Chokeberry	Bilberry	Elderberry
C*	day 0	17.44 ± 0.02	14.40 ± 0.02	9.99 ± 0.02
	day 28	14.72 ± 0.01 ^a^	14.21 ± 0.02 ^a^	8.93 ± 0.04 ^a^
*h°*	day 0	15.03 ± 0.09	13.71 ± 0.06	14.26 ± 0.01
	day 28	13.97 ± 0.05 ^a^	12.82 ± 0.06	11.55 ± 0.26 ^a^
∆*E* ^b^		3.55 ± 0.03 ^A^	0.38 ± 0.05 ^C^	2.32 ± 0.19 ^B^

^a^: Value at day 28 is significantly different compared to the value at day 0. ^b^: Different letters indicate significant differences (*p* ≤ 0.05) within the row.

**Table 5:** Total phenolic content of yogurt applications containing chokeberry, bilberry, and elderberry PP and purple sweet potato anthocyanins (PSP) and black carrot concentrate (BC) stored for 14 days with expression of values of day 0 and day 14. Values are mean ± standard deviation (*n* = 3).

	Total Phenolic Content (mg GAE/100 g fw Yogurt)	
	Day 0	Day 14
Chokeberry PP	71.27 ± 2.85	73.45 ± 2.49
PSP reference chokeberry	23.62 ± 4.66	29.68 ± 7.13
BC reference chokeberry	17.24 ± 4.66	29.75 ± 7.13
Bilberry PP	71.97 ± 11.43	65.14 ± 9.09
PSP reference bilberry	25.09 ± 3.33	17.32 ± 0.71
BC reference bilberry	21.54 ± 2.50	11.21 ± 0.80
Elderberry PP	65.38 ± 8.47	85.75 ± 4.15 ^a^
PSP reference elderberry	18.91 ± 3.09	37.17 ± 12.41
BC reference elderberry	10.92 ± 4.54	17.27 ± 5.71

^a^: Value at day 14 is significantly different compared to the value at day 0.

**Table 6:** Color parameters, namely color difference ∆*E*, Chroma C*, and hue angle *h°* of yogurt applications containing chokeberry, bilberry, and elderberry PP and purple sweet potato anthocyanins (PSP) and black carrot (BC) over storage time and day 0 and day 14.

	∆*E*	*h°*		C*	
		Day 0	Day 14	Day 0	Day 14
Chokeberry PP	1.87	1.79	0.46	19.74	19.68
PSP reference chokeberry	3.72	12.92	10.37	19.61	17.39
BC reference chokeberry	1.96	1.75	0.70	20.29	19.11
Bilberry PP	2.17	10.42	9.98	16.78	16.30
PSP reference bilberry	2.94	10.05	7.62	16.27	14.83
BC reference bilberry	2.61	0.75	2.97	16.04	14.90
Elderberry PP	4.89	3.59	4.2	15.16	15.61
PSP reference elderberry	5.72	9.20	4.20	15.50	13.17
BC reference elderberry	2.94	2.79	5.35	14.33	13.30

**Table 7:** Amount of reference substance (%), purple sweet potato anthocyanins, and black carrot concentrate used for the adjustment of color saturation of each yogurt application from the three PP.

	Chokeberry	Bilberry	Elderberry
Purple sweet potato anthocyanins (%)	0.43	0.33	0.27
Black carrot concentrate (%)	0.32	0.18	0.14

## Data Availability

Not applicable.

## Supplementary Materials

The following are available online, Figure S1: UHPLC-DAD chromatogram (520 nm) of the pomace powder extracts: (A) chokeberry, (B) bilberry, and (C) elderberry from the untreated pomace (black line), the pomace powder on day 0 (grey line), and on day 28 (dotted line). Peak classification is shown in the Table., Figure S2: UHPLC-DAD chromatogram (520 nm) of yoghurt applications fortified with 2% (*w/w*) pomace powder: (A) chokeberry, (B) bilberry, and (C) elderberry on day 0 (black line), and on day 14 (dotted line). Peak classification is shown in the Table., Table S1: Dry matter (%) of the raw material (untreated berry pomace) and the mass balance (kg) for the drying and milling process of the three pomaces with corresponding residual moisture content (%) of the dried pomace as well as the total anthocyanin content (mg Cya-Glc eq./100g dw) of the untreated berry pomace., Table S2: Total anthocyanin content (g Cya-Glc eq./100g dw) of the reference substances for PP storage experiments, namely XAD7 extracts of chokeberry and bilberry and their corresponding purified anthocyanins.

## Sample Availability

Samples of the compounds are not available from the authors.
