# Supplementary material for: Application of Crude Pomace Powder of Chokeberry, Bilberry, and Elderberry as a Coloring Foodstuff
Source: Molecules. 2021 May 4;26(9):2689. doi: 10.3390/molecules26092689 (PMC8124194; doi:10.3390/molecules26092689)
Supplement: Supplementary file 1 [file molecules-26-02689-s001.zip › Supplementary_information_new.pdf]

Table S1. Dry matter (%) of the raw material (untreated berry pomace) and the mass balance (kg) for the drying and milling process of the three berry pomaces with corresponding residual moisture content (%) of the dried pomace as well as the total anthocyanin content (mg Cya-Glc eq./100 g dw) of the untreated berry pomace.

	**chokeberry**	**bilberry**	**elderberry**
dry matter content of untreated pomace (%)	54.63 ± 2.54	50.58 ± 0.99	57.07 ± 0.89
dry matter content of dried pomace (%)	95.24 ± 1.52	93.31 ± 1.84	92.81 ± 0.94
amount of untreated pomace (kg)	5.23	4.52	7.23
amount of powder from seedless fraction (kg)	0.50	0.23	0.17
total anthocyanin content of untreated pomace (mg Cya-Glc eq./100g dw)*	2188.13 ± 177.42	1966.79 ± 121.97	196.72 ± 6.36

*: preliminary experiments showed no considerable degradation of anthocyanins during the drying process.

Table S2. Total anthocyanin content (g Cya-Glc eq./100 g dw) of the reference substances for PP storage experiments, namely XAD7 extracts of chokeberry and bilberry and their corresponding purified anthocyanins.

	total anthocyanin content (g Cya-Glc eq./100 g dw)	
	day 0	day 28
bilberry XAD7 extract	22.32	43.65
bilberry anthocyanins	49.49	79.24
chokeberry XAD7 extract	6.75	8.07
chokeberry anthocyanins	26.68	23.57

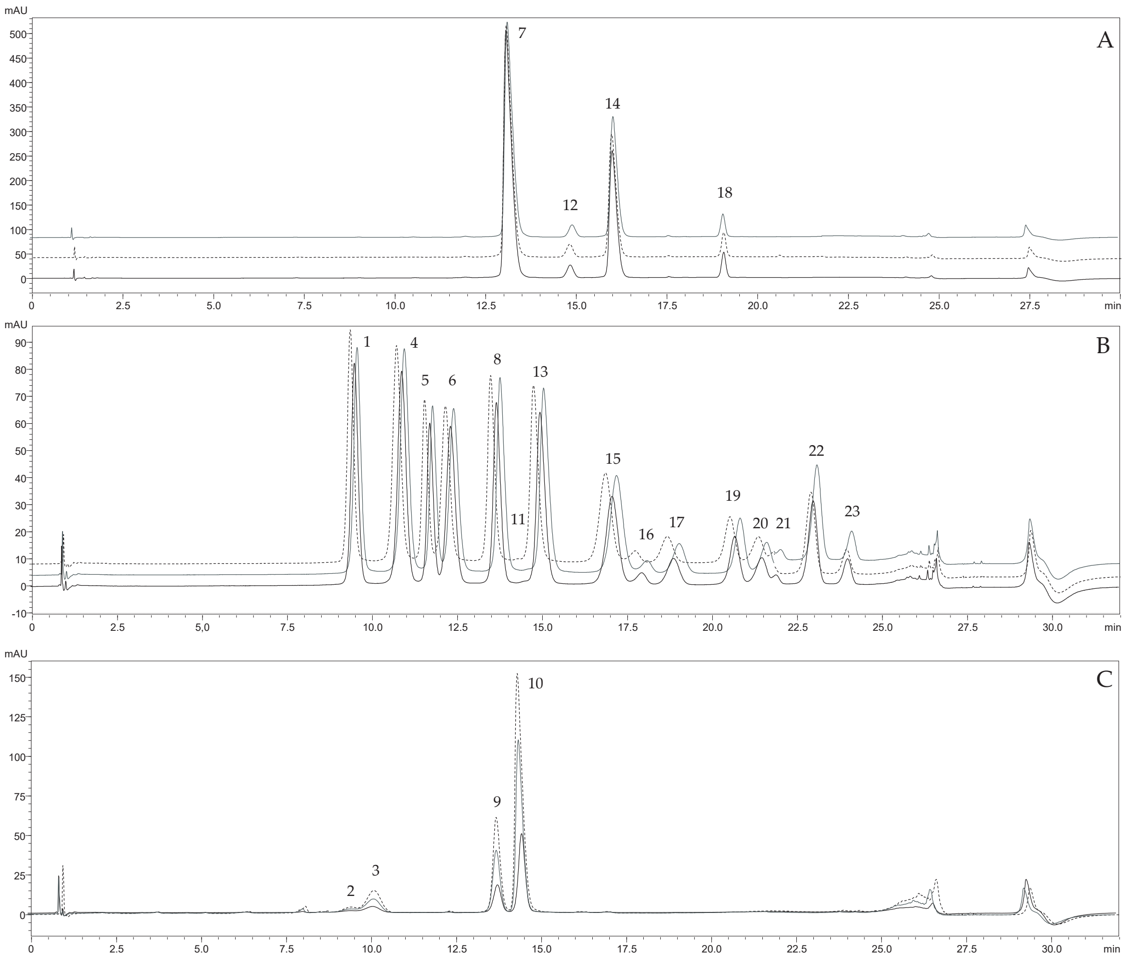

no	compound	ret time	reference
1	Del-3-gal	9,505	[43]
2	Cya-3-sam-5-glu	9,532	[44]
3	Cya-3,5-diglu	10,177	[44]
4	Del-3-glu	10,902	[43]
5	Cya-3-gal	11,737	[43]
6	Del-3-ara	12,343	[43]
7	Cya-3-gal	13,160	[44]
8	Cya-3-glu	13,702	[43]
9	Cya-3-sam	13,816	[44]
10	Cya-3-glu	14,480	[44]
11	Pet-3-gal	14,547	[43]
12	Cya-3-glu	14,947	[44]
13	Cya-3-ara	14,963	[43]
14	Cya-3-ara	16,074	[44]
15	Pet-3-glu	17,085	[43]
16	Peo-3-gal	17,976	[43]
17	Pet-3-ara	18,915	[43]
18	Cya-3-xyl	19,105	[44]
19	Peo-3-glu	20,719	[43]
20	Mal-3-gal	21,509	[43]
21	Peo-3-ara	21,920	[43]
22	Mal-3-glu	23,019	[43]
23	Mal-3-ara	24,038	[43]

Figure S1. UHPLC-DAD chromatogram (520 nm) of the pomace or pomace powder extracts: (A) chokeberry, (B) bilberry, and (C) elderberry from the untreated pomace (black line), the pomace powder on day 0 (grey line), and on day 28 (dotted line). Peak classification is shown in the Table.

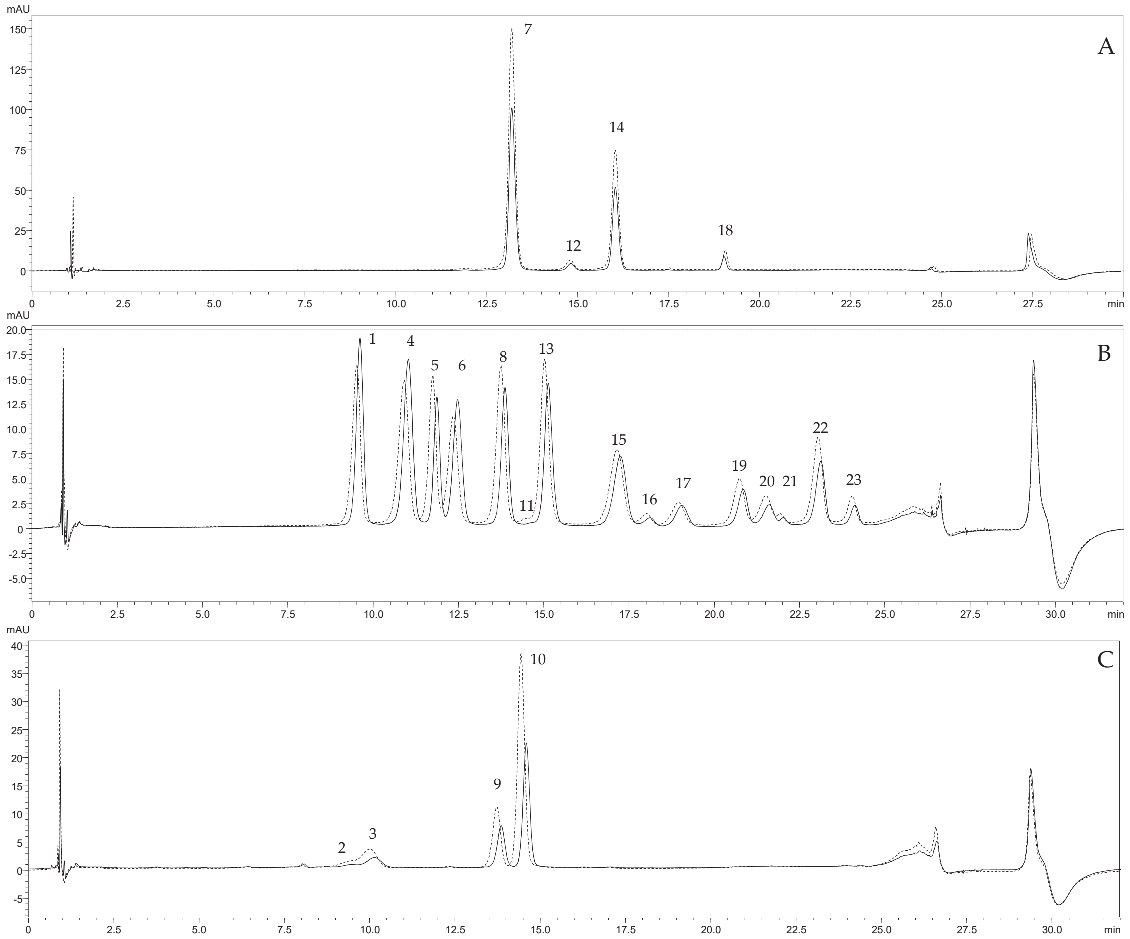

no	compound	ret time	reference
1	Del-3-gal	9,505	[43]
2	Cya-3-sam-5-glu	9,532	[44]
3	Cya-3,5-diglu	10,177	[44]
4	Del-3-glu	10,902	[43]
5	Cya-3-gal	11,737	[43]
6	Del-3-ara	12,343	[43]
7	Cya-3-gal	13,160	[44]
8	Cya-3-glu	13,702	[43]
9	Cya-3-sam	13,816	[44]
10	Cya-3-glu	14,480	[44]
11	Pet-3-gal	14,547	[43]
12	Cya-3-glu	14,947	[44]
13	Cya-3-ara	14,963	[43]
14	Cya-3-ara	16,074	[44]
15	Pet-3-glu	17,085	[43]
16	Peo-3-gal	17,976	[43]
17	Pet-3-ara	18,915	[43]
18	Cya-3-xyl	19,105	[44]
19	Peo-3-glu	20,719	[43]
20	Mal-3-gal	21,509	[43]
21	Peo-3-ara	21,920	[43]
22	Mal-3-glu	23,019	[43]
23	Mal-3-ara	24,038	[43]

Figure S2. UHPLC-DAD chromatogram (520 nm) of yoghurt applications fortified with 2% (*w/w*) pomace powder: (A) chokeberry, (B) bilberry, and (C) elderberry on day 0 (black line), and on day 14 (dotted line). Peak classification is shown in the Table.
